# Fine Particulate Matter (PM_2.5_) Air Pollution and Selected Causes of Postneonatal Infant Mortality in California

**DOI:** 10.1289/ehp.8484

**Published:** 2006-01-13

**Authors:** Tracey J. Woodruff, Jennifer D. Parker, Kenneth C. Schoendorf

**Affiliations:** 1 Office of Policy, Economics, and Innovation, U.S. Environmental Protection Agency, San Francisco, California, USA; 2 National Center for Health Statistics, Hyattsville, Maryland, USA

**Keywords:** air pollution, infant mortality, particulate matter air pollution, PM_2.5_, postneonatal

## Abstract

Studies suggest that airborne particulate matter (PM) may be associated with postneonatal infant mortality, particularly with respiratory causes and sudden infant death syndrome (SIDS). To further explore this issue, we examined the relationship between long-term exposure to fine PM air pollution and postneonatal infant mortality in California. We linked monitoring data for PM ≤2.5 μm in aerodynamic diameter (PM_2.5_) to infants born in California in 1999 and 2000 using maternal addresses for mothers who lived within 5 miles of a PM_2.5_ monitor. We matched each postneonatal infant death to four infants surviving to 1 year of age, by birth weight category and date of birth (within 2 weeks). For each matched set, we calculated exposure as the average PM_2.5_ concentration over the period of life for the infant who died. We used conditional logistic regression to estimate the odds of postneonatal all-cause, respiratory-related, SIDS, and external-cause (a control category) mortality by exposure to PM_2.5_, controlling for the matched sets and maternal demographic factors. We matched 788 postneonatal infant deaths to 3,089 infant survivors, with 51 and 120 postneonatal deaths due to respiratory causes and SIDS, respectively. We found an adjusted odds ratio for a 10−μg/m^3^ increase in PM_2.5_ of 1.07 [95% confidence interval (CI), 0.93–1.24] for overall postneonatal mortality, 2.13 (95% CI, 1.12–4.05) for respiratory-related postneonatal mortality, 0.82 (95% CI, 0.55–1.23) for SIDS, and 0.83 (95% CI, 0.50–1.39) for external causes. The California findings add further evidence of a PM air pollution effect on respiratory-related postneonatal infant mortality.

High air pollution levels have been linked to infant mortality. An early example is the London Fog episode of 1952, where a sharp increase in particulate matter (PM) air pollution led to increased mortality among infants and older adults (Her Majesty’s Public Health Service 1954). Contemporary studies in countries with relatively high PM air pollution levels have also found an association between infant mortality and PM air pollution (Bobak and Leon 1999; Ha et al. 2003; Loomis et al. 1999). These studies further suggest that PM air pollution is associated with postneonatal mortality (deaths occurring after 28 days of life), with respiratory causes having the highest association (Bobak and Leon 1999; Ha et al. 2003). However, these countries have relatively high ambient air pollution compared with concentrations in North America. A study using U.S. data from 1989 through 1991 found that longer-term exposure to PM air pollution was associated with postneonatal deaths attributable to respiratory-related causes and sudden infant death syndrome (SIDS) (Woodruff et al. 1997). A follow-up study examining annual concentrations of PM air pollution in 1990 confirmed the original analysis and also found that carbon monoxide was not related to infant mortality (Lipfert et al. 2000). However, a study in Canada found that short-term increases in nitrogen dioxide and sulfur dioxide, and not PM air pollution, were associated with SIDS between 1984 and 1999 (Dales et al. 2004). Recent reviews of infant mortality and air pollution have suggested that air pollution seems to be most consistently associated with postneonatal mortality from respiratory causes and possibly SIDS, but further research is needed to clarify these relationships (Glinianaia et al. 2004; Tong and Colditz 2004).

Until recently, studies evaluating the relationship between air pollution and mortality in adults and infants have focused on PM ≤ 10 μm in aerodynamic diameter (PM_10_). Since the late 1980s, this PM has been the focus of health studies because it is respirable. However, studies over the last several years suggest that it may be the smaller PM, measured at ≤2.5 μm in aerodynamic diameter (PM_2.5_) that is often more likely to be associated with these health effects [Laden et al. 2000; Schwartz and Neas 2000; U.S. Environmental Protection Agency (EPA) 2004]. PM_2.5_ may be more toxic because most of this PM fraction is from combustion sources, such as cars, utilities, and wood burning, rather than typical crustal sources, such as road dust and agricultural fields (U.S. EPA 2004).

Further analysis is warranted to investigate the possible link between air pollution and infant mortality in the United States. Using a matched case–control design, we examined the potential relationship between PM_2.5_ and plausible causes of postneonatal mortality in California in 1999–2000 based on a unique data set, which allows estimating individual long-term exposure to PM_2.5_. In addition, we evaluated these results in light of previous work on infant mortality and PM air pollution in the United States (Lipfert et al. 2000; Woodruff et al. 1997).

## Materials and Methods

### Study population.

Linked birth and infant death records were obtained from the California Department of Health Services for births occurring in 1999 and 2000 (California Department of Health Services 1999–2000). We limited our study population to singleton births. We excluded the 6,335 (0.6%) births with missing data for one or more of the following covariates: maternal race, marital status, parity, maternal education, and maternal age. These variables were used to compare our study population with the overall population of births as well as to control for the potentially confounding effects of demographic factors on the association between infant mortality and air pollution. Maternal race and Hispanic origin were collapsed into a single categorical variable with five levels (African American, Asian, Mexican, white, and other); there were too few mothers in other groups to examine separately. Mothers who were Hispanic but not Mexican were categorized by race rather than included with the Mexican mothers because there are large differences in infant mortality among Hispanic subgroups (Mathews et al. 2003), and the vast majority of Hispanic mothers in California are Mexican (87% in 2000).

Finally, to consider only those deaths more plausibly associated with air pollution, we limited our study to infant deaths occurring 28 days after birth (postneonatal); deaths before that time are more likely to occur in infants who had not left the hospital after birth or from complications attributable to pregnancy. After these exclusions, 1,606 postneonatal infant deaths and 1,010,054 survivors remained eligible for inclusion in our study.

### Exposure assessment.

Air pollution monitoring data for 1999 through 2001 were obtained from the California Air Resources Board for PM_2.5_ (California Air Resources Board 2003). PM_2.5_ was measured continuously for 24 hr every 6 days. Monitors specifically designed to collect background concentrations or source specific concentrations of PM_2.5_ were excluded, as were monitors with < 45 measurements in a year. This study used data from 73 monitors located in 39 counties.

To calculate exposures for our study group, we used information on maternal address of residence as reported on the birth certificate. Only infants with known maternal addresses within 5 miles of a PM_2.5_ monitor were included in the analysis. The distance between maternal residence at the time of delivery and each of the monitors was computed using the latitude and longitude of all locations. The distance values were used to identify the nearest monitor within 5 miles of each mother’s residence. Exposure estimates for each infant were based on that nearest 5-mile monitor.

After limiting our study population to births to mothers who lived within 5 miles of a PM_2.5_ monitor, 788 postneonatal deaths and 468,448 eligible survivors remained for our analysis.

### Matching.

We divided the infants into seven birth weight categories (< 1,500, 1,500–1,999, 2,000–2,499, 2,500–2,999, 3,000–3,499, 3,500–3,999, and ≥4,000 g). Each postneonatal death was randomly matched to four infants who survived to 1 year of age. The infants were matched by date of birth (within 2 weeks) and birth weight category. Because infant mortality depends on birth weight, we matched by birth weight category to control for potential confounding by birth weight. For example, if an infant who died was born on 1 February and weighed 2,600 g, we matched this infant to four other randomly selected infants born between 18 January and 15 February who weighed between 2,500 and 2,999 g and lived to 1 year of age. We matched the 788 postneonatal infant deaths to 3,152 infant survivors, giving us a study population of 3,940.

To obtain a measure of long-term exposure to PM_2.5_, we calculated the average air pollution levels for the time period between birth and the postneonatal death for the infant who died and for the same period for its four matched surviving infants. Using the example above, if the infant born on 1 February died on 30 March, we calculated the average PM_2.5_ levels from the nearest monitor within 5 miles between 1 February and 30 March for this infant and the four matched surviving infants. This allowed us to compare the air pollution levels between the deceased and surviving infants up until the time of death. Ninety-eight percent of California infants and 97% of postneonatal infant deaths were born in counties with PM_2.5_ monitors. We also assessed the sensitivity of the relationship to two other more commonly available exposure metrics, because public natality data sets do not include maternal addresses or date of birth. One exposure metric used the matched exposure period between birth and death but averaged all PM_2.5_ measurements in the mother’s county. The second exposure metric was based on the average of all PM_2.5_ measurements in the mother’s county over the 2-year study using the period 2000 and 2001 as representative of the average exposure of the period.

Of the 3,152 matched survivors, 63 were excluded because they did not have any measurements within the reference time period with which to calculate an exposure. This final exclusion left 3,877 infants for our analysis.

### Analysis.

We used conditional logistic regression to estimate the odds of all-cause and cause-specific postneonatal mortality by exposure to air pollution, controlling for the matched sets (Stata version 8; StataCorp 2003). *International Classification of Diseases, 10th Revision* (ICD-10) (World Health Organization 1993) codes for the underlying cause of death were obtained from the death certificate information included in the linked birth and death records. SIDS was defined as ICD-10 code R95. Respiratory mortality primarily included underlying cause of death codes (J00–J99), plus deaths coded P27.1 [bronchopulmonary dysplasia (BPD)] (World Health Organization 1993). Deaths from respiratory causes not likely to be influenced by air pollution (e.g., J69.0, aspiration pneumonia) were not included as “respiratory deaths” in this analysis. A complete list of ICD-10 codes is available from the authors. In addition, we looked at infants who died from external causes (e.g., accidents, ICD-10 codes V01–Y98) where we would not expect any association with air pollution. Finally, we further evaluated the association between PM_2.5_ and mortality from BPD, as well as the association with respiratory death exclusive of BPD. BPD represents the single most commonly reported cause of death among the infants who died of respiratory-related causes (25%). Infants with BPD may have particular susceptibility to PM, because these infants are most often born prematurely and have underlying pulmonary pathology.

We modeled air pollution exposure using a continuous, linear form. Additional forms of the exposure variables were examined, including natural log-transformation, inclusion of squared term, and use of quartiles (data not shown). We included maternal race, education, parity, age, and marital status in the regression models to obtain adjusted estimates. Continuous forms of education, parity, and age were used in the final models, although categorical forms were examined to assess their effect on the PM–mortality relationships (data not shown). Because there was no difference between categorical and continuous forms, we used the continuous form to create a more parsimonious model. In the models, for mother’s race, the few births to American-Indian mothers and to mothers reporting some other race were included in the reference group with the white mothers.

## Results

Of the 788 infant deaths in our data set, there were 51 deaths from respiratory causes (13 from BPD), 136 deaths from SIDS, and 55 deaths from external causes. Compared with the infants who died, the demographic characteristics of the surviving infants were similar for most measures (Table 1). There were slightly fewer married women, slightly fewer first-time births, slightly more mothers with < 12 years of education, and more African-American mothers among the infants who died than among the matched survivors. The demographic characteristics of mothers of the surviving infants were similar to those of eligible births in California. Of the 788 infant deaths in our data set, 46% died between 1 and 3 months of age, 30% died between 3 and 6 months of age, and 24% died after 6 months of age.

The median PM_2.5_ concentration was slightly higher among the infants who died of all causes and of respiratory causes compared with the surviving infants (Table 2). The median PM_2.5_ concentrations were slightly lower for the infants who died of SIDS and of external causes compared with their matched survivors (Table 2).

Table 3 shows the adjusted and unadjusted odds ratios (ORs) for a 10−μg/m^3^ increase in PM_2.5_ for overall and for cause-specific postneonatal infant mortality. In general, adjusting for maternal characteristics slightly decreased the ORs. The adjusted OR for overall mortality was 1.07 [95% confidence interval (CI), 0.93–1.24] for a 10−μg/m^3^ increase in PM_2.5_. There was a stronger relationship with postneonatal respiratory deaths, with an adjusted OR of 2.13 (95% CI, 1.12–4.05) for a 10−μg/m^3^ increase in PM_2.5_. There was no association with external causes (adjusted OR for a 10−μg/m^3^ increase in PM_2.5_, 0.83; 95% CI, 0.50–1.39).

We evaluated the sensitivity of the association between respiratory mortality and PM_2.5_. First, we excluded the 13 deaths due to BPD and their matched survivors. This resulted in an adjusted OR of 1.42 (95% CI, 0.66–3.03). For the small group of BPD deaths, the unadjusted OR was 6.00 (95% CI, 1.40–27.76); too few infants were in this group to obtain stable adjusted estimates. Because, as expected, nearly all of these BPD infants had low birth weight (< 2,500 g), we further examined the association between respiratory death and PM_2.5_ by birth weight. We found a stronger association among the 23 low-birth-weight infants who died of any respiratory cause (unadjusted OR, 3.09; 95% CI, 1.14–8.40) than among the 28 normal-birth-weight infants (≥2,500 g) who died of respiratory causes (unadjusted OR, 1.66; 95% CI, 0.74–3.70); as with the BPD analysis, too few infants were in these subgroups to obtain adjusted estimates.

We did not find a relationship between PM_2.5_ and SIDS (adjusted OR, 0.82; 95% CI, 0.55–1.23) for a 10−μg/m^3^ increase in PM_2.5_ (Table 3). We further stratified the SIDS matched sets by season to account for potential seasonal effects, but in part because of lack of power, there was no significant difference in the association between PM_2.5_ and SIDS by season of birth.

Because the observed SIDS relationship was much different than previous analyses, which found a statistically significant relationship between SIDS and PM_10_ (Woodruff et al. 1997), we further explored possible explanations. Since 1990, the diagnostic requirements of SIDS have become more rigorous. (Willinger et al. 1991), and a recent analysis has suggested that between 1999 and 2001, there has been a shift in diagnosis from SIDS to other non-SIDS causes (Malloy and MacDorman 2005). Malloy and MacDorman (2005) identify ICD-10 code R99, “ill-defined and unspecified causes of mortality,” as one of the categories that may be absorbing some of the shift from the SIDS code. Including R99 with R95 (SIDS), we found an adjusted OR for a 10− μg/m^3^ increase in PM_2.5_ of 1.03 (95% CI, 0.79–1.35), compared with 0.82 (95% CI, 0.55–1.23) for R95 alone. A second possibility is a difference in the type of PM exposure, because previous analyses assessed PM_10_ and this analysis used PM_2.5_. We examined the relationship between PM_10_ and SIDS for those infants that could be linked to both a PM_10_ monitor and a PM_2.5_ monitor within 5 miles of the maternal residence. We did not find any relationship between SIDS and PM_10_ or the coarse particles (PM_10_–PM_2.5_) (data not shown).

Additional models for all outcomes were fit using a natural log-transformation of the exposure as well as the exposure squared. However, a simple examination of the *Z*-statistics and likelihood ratio statistics did not indicate that these models were better than the linear form of PM_2.5_ (data not shown). Models with PM_2.5_ categorized into quartiles showed increasing associations with respiratory mortality, but much larger CIs. When compared with PM_2.5_ exposure in the lowest quartile, those respiratory deaths in the highest quartile had an adjusted OR of 2.35 (95% CI, 0.85–6.54); those respiratory deaths in the third quartile, 1.75 (95% CI, 0.65–4.72); and those respiratory deaths in the second, 1.28 (95% CI, 0.47–3.51). For all-cause mortality and SIDS, there was no apparent relationship with PM_2.5_ using quartiles of exposure (data not shown).

A comparison of the two different exposure metrics (matched PM_2.5_ averaged over all monitors in the county and averaging all PM_2.5_ measurements in the county over the 2-year study period) found results similar to those presented. For example, for respiratory deaths, the adjusted OR for a 10−μg/m^3^ increase in PM_2.5_ calculated averaging all measurements in the county collected over the matched exposure period was 2.28 (95% CI, 0.94–5.52). The adjusted OR for a 10−μg/m^3^ increase in PM_2.5_ as the average of all measurements in the county over the 2-year study period was 2.26 (95% CI, 0.83–6.21). This is similar to the PM_2.5_ matched exposure metric shown in Table 3 [adjusted OR, 2.13 (95% CI, 1.12–4.05) for a 10−μg/m^3^ increase in PM_2.5_] but with wider CIs.

## Discussion

In this analysis we found a relationship between postneonatal mortality from respiratory causes and long-term exposure to PM_2.5_ but not between mortality from external causes, such as accidents and homicides, and PM_2.5_ in California. These findings are consistent with suspected mechanisms of the effect of PM on infant health. However, unlike some prior analyses using vital statistics data, we did not find an association between PM and SIDS.

Among the respiratory deaths, the relationship was stronger among the low-birth-weight infants, in general, as well as among those with BPD as an underlying cause of death. This suggests that these infants, and perhaps others with underlying lung conditions, may be at higher risk of susceptibility to air pollution. However, these results will need further assessment, because the small number of cases limited our ability to fully evaluate these findings. In addition, infants with BPD often remain in the hospital for a substantial period. We have no information on whether the infant was discharged from the hospital before death, nor is there an indication of whether those infants were breathing room air or were receiving supplemental oxygen.

This analysis in California attempts to build upon previous analyses in the United States in several ways. First, in this analysis, we use the exposure most relevant to the infant deaths, which occurs between the birth and the death of the infant, and compare it with the same exposure window among the surviving infants. This eliminates exposures outside this temporal window, because exposures before the infant’s birth and after its death would not contribute to the direct risk of postneonatal mortality. Second, other studies suggest that air pollution is linked to low birth weight and preterm birth, which could indirectly affect the risk of infant mortality (Maisonet et al. 2001; Parker et al. 2005; Ritz and Yu 1999; Ritz et al. 2000). In this analysis, we attempt to focus on the direct effects of pollution on infant mortality by matching on birth weight and by limiting the study to postneonatal deaths.

Third, we use the addresses of the mothers to limit our study population to mothers who live within 5 miles of a PM_2.5_ monitor. Although we do not know whether this restriction provides a better measure of the infants’ exposures, particularly because PM_2.5_ has been shown to be evenly distributed across some large geographic areas (U.S. EPA 2004; Wilson et al. 2000), this residential detail allowed for a comparison of a potentially more precise measure than the county-level measure more commonly employed. It should be noted that the PM_2.5_ is generally measured every 6 days, and episodes of high concentrations occurring between measurements would not be reflected in the average, possibly leading to some exposure misclassification.

We also use outdoor monitors to represent exposure to the infants. Studies show that outdoor monitors provide a reasonable estimate of exposure to ambient concentrations of PM and that exposures to ambient concentrations are highly correlated to outdoor air monitors (Wilson et al. 2000).

In this analysis, we did not find a relationship between SIDS and PM_2.5_ in California. A previous analysis by Woodruff et al. (1997) found a relationship between SIDS and PM_10_ for infants born between 1989 and 1991 in 86 cities across the United States. In a later reanalysis of similar data, Lipfert et al. (2002) found a comparable relationship between PM_10_ and SIDS for infants born in 1990 in the United States. However, a recent qualitative review of nine studies of air pollution and SIDS concluded that the current evidence is inadequate to fully assess the relationship but that the evidence is suggestive (Tong and Colditz 2004). There are several possible explanations for the difference in the results. One is that the original findings were spurious, although two different studies using similar data sets found similar relationships (Lipfert et al. 2000; Woodruff et al. 1997). The Lipfert et al. (2002) analysis suggested possible geographic variability in the association, however. California was not included in the Woodruff et al. (1997) analysis. In addition, an analysis of 12 Canadian cities between 1984 and 1999 did not find daily variation in PM_10_ to be associated with SIDS, although they did find an association with more acute exposures to SO_2_ and NO_2_ (Dales et al. 2004). However, these authors note that they have relatively little PM_2.5_ data in their analysis and possibly did not see an effect due to lack of statistical power. We also assessed whether birth weight was an important factor in the SIDS findings, but stratifying by low birth weight and normal birth weight for SIDS deaths did not change the results. Finally, we did not find a relationship between SIDS and PM_10_ or coarse particles.

Since the early 1990s, increased attention to SIDS deaths and promulgation of stricter criteria for diagnosis of SIDS may have also changed the composition of infant deaths diagnosed as SIDS (Malloy and MacDorman 2005). For instance, a death scene investigation is now considered an integral part of a SIDS diagnosis (Willinger et al. 1991). A recent analysis of trends in SIDS and other related causes of postneonatal infant mortality found that increases from unknown and unspecified causes and suffocation account for 90% of the decrease in SIDS rate between 1999 and 2001 (Malloy and MacDorman 2005). This suggests that there may have been a shift in diagnosis of SIDS deaths. Previous analyses of infant mortality and PM_10_ investigated SIDS as a cause of death because it has been associated with exposure to environmental tobacco smoke, which is also an air pollutant, and because SIDS may be related to cardiovascular or respiratory events, which could be influenced by PM air pollution exposure. Finally, it could be that the composition of PM_2.5_ in California is different than in other locations in the United States. There is evidence to suggest that nitrates compose a larger fraction of PM_2.5_ in California than in other regions, that there is more local contribution to PM_2.5_ than regional, and that the predominant sources of PM_2.5_ are from mobile sources and agriculture, whereas it is a mix of mobile and stationary sources elsewhere (McMurry et al. 2004). Further investigation is needed to understand why the findings from these studies differ.

We adjusted for a number of maternal demographic factors, although the effect of the adjustments on the estimated ORs for respiratory and SIDS deaths was minimal. We were not able to control for maternal smoking, because it is not available on the California birth certificates. However, this is unlikely to significantly affect the estimates, because our previous analysis of PM air pollution and infant mortality did not find smoking to significantly alter the association between infant mortality and air pollution (Woodruff et al. 1997).

The respiratory results from this study are consistent with recent similar analyses in the Czech Republic and South Korea (Bobak and Leon 1999; Ha et al. 2003). The Czech Republic study, which uses the same matched study design as our analysis, for postneonatal infant mortality gives an adjusted odds ratio of 1.12 (95% CI, 0.96–1.30) for total mortality and 1.45 (95% CI, 1.01–2.10) for respiratory-related mortality for an interquartile increase in total suspended particulates (Bobak and Leon 1999. They did not find an association for nonrespiratory-related postneonatal infant mortality. Similarly, in the South Korea, using a time-series approach for postneonatal infant mortality gives an adjusted relative risk of 1.13 (95% CI, 1.09–1.18) for total mortality and 2.03 (95% CI, 1.79–2.31) for respiratory mortality for an interquartile increase in PM_10_ (Ha et al. 2003). Our analysis in California finds an adjusted OR of 1.07 (95% CI, 0.93–1.24) for total and 1.75 (95% CI, 1.09–2.82) for respiratory-related mortality for an interquartile increase in PM_2.5_.

This work shows an association between respiratory-related postneonatal mortality and fine PM air pollution in California. This adds further evidence to the previous literature in the United States and in other countries that air pollution may be associated with some portion of infant mortality in the United States.

## Figures and Tables

**Table 1:** Characteristics of births in study sample^a^ and overall population of singleton infants: California 1999–2000.

Maternal factors	Postneonatal deaths (*n* = 788)	Matched survivors (*n* = 3,089)	Eligible births in California^b^ (*n* = 1,014,752)
Age (mean years)	26	27	28
Married (%)	56	65	69
Parity (% first birth)	36	42	39
Education (%)			
< 12 years	44	35	31
12 years	30	30	29
13–15 years	17	19	20
≥ 16 years	9	16	20
Race and Hispanic origin (%)^c^			
Asian	11	13	12
African American	17	9	7
Mexican American	42	47	43
White	30	31	38
Other race or ethnicity	1	1	1

a Study sample consists of singleton infants who lived within 5 miles of PM_2.5_ monitor either who died in the postneonatal period or who were matched survivors, with information for maternal age, maternal race, maternal education, parity, and birth weight.

b All singleton births to residents of California with information on maternal age, race, education, parity, and infant birth weight were initially eligible for the study.

c Because of rounding, the numbers may not add up to exactly 100%.

**Table 2:** Median (interquartile range) of PM_2.5_ concentrations (μg/m^3^) for total and cause-specific post neonatal infant deaths and matched infant survivors in California 1999–2000.

	Deaths		Matched survivors	
	No.	PM_2.5_	No.	PM_2.5_
All causes	788	19.2 (13.4–23.6)	3,089	18.4 (13.5–22.7)
Respiratory	51	19.8 (16.0–23.4)	201	17.9 (13.5–22.3)
SIDS	136	17.6 (11.1–23.6)	533	18.1 (13.2–22.0)
External causes	55	17.3 (12.3–23.9)	220	18.6 (13.7–23.0)

**Table 3:** ORs (95% CIs) for total and cause-specific postneonatal mortality and PM_2.5_^a^ in California, 1999–2000.

Cause of postneonatal death	Unadjusted	Adjusted for maternal characteristics^b^
All causes	1.15 (1.00–1.32)	1.07 (0.93–1.24)
Respiratory	2.15 (1.15–4.02)	2.13 (1.12–4.05)
SIDS	0.86 (0.61–1.22)	0.82 (0.55–1.23)
External causes	0.91 (0.56–1.47)	0.83 (0.50–1.39)

a OR for a 10−μg/m^3^ increase in PM_2.5_.

b Adjusted for maternal race, education, age, marital status, and parity.
